# Minimalist Deployment of Neural Network Equalizers in a Bandwidth-Limited Optical Wireless Communication System with Knowledge Distillation

**DOI:** 10.3390/s24051612

**Published:** 2024-03-01

**Authors:** Yiming Zhu, Yuan Wei, Chaoxu Chen, Nan Chi, Jianyang Shi

**Affiliations:** 1 Key Laboratory for Information Science of Electromagnetic Waves (MoE), Department of Communication Science and Engineering, Fudan University, Shanghai 200433, China; 23210720341@m.fudan.edu.cn (Y.Z.); 23210720068@m.fudan.edu.cn (Y.W.); 23110720110@m.fudan.edu.cn (C.C.); nanchi@fudan.edu.cn (N.C.); 2 Shanghai Engineering Research Center of Low-Earth-Orbit Satellite Communication and Applications, Shanghai 200433, China; 3 Shanghai Collaborative Innovation Center of Low-Earth-Orbit Satellite Communication Technology, Shanghai 200433, China

**Keywords:** artificial intelligence, machine learning, neural network, knowledge distillation, nonlinear equalizer, biGRU, 1D-CNN

## Abstract

An equalizer based on a recurrent neural network (RNN), especially with a bidirectional gated recurrent unit (biGRU) structure, is a good choice to deal with nonlinear damage and inter-symbol interference (ISI) in optical communication systems because of its excellent performance in processing time series information. However, its recursive structure prevents the parallelization of the computation, resulting in a low equalization rate. In order to improve the speed without compromising the equalization performance, we propose a minimalist 1D convolutional neural network (CNN) equalizer, which is reconverted from a biGRU with knowledge distillation (KD). In this work, we applied KD to regression problems and explain how KD helps students learn from teachers in solving regression problems. In addition, we compared the biGRU, 1D-CNN after KD and 1D-CNN without KD in terms of Q-factor and equalization velocity. The experimental data showed that the Q-factor of the 1D-CNN increased by 1 dB after KD learning from the biGRU, and KD increased the RoP sensitivity of the 1D-CNN by 0.89 dB with the HD-FEC threshold of 1 × 10^−3^. At the same time, compared with the biGRU, the proposed 1D-CNN equalizer reduced the computational time consumption by 97% and the number of trainable parameters by 99.3%, with only a 0.5 dB Q-factor penalty. The results demonstrate that the proposed minimalist 1D-CNN equalizer holds significant promise for future practical deployments in optical wireless communication systems.

## 1. Introduction

In recent years, optical wireless communication (OWC) has become a big focus of next-generation 6G communication [1]. As a branch of it, free-space optical communication (FSO) has many advantages such as easy deployment, high bandwidth and strong security. However, in practical applications, geometric loss, pointing error, atmospheric attenuation and turbulence effects will affect the signal transmission, and the atmospheric turbulence is particularly obvious. These effects will cause the signal to produce more serious distortions. In addition, the transmission of carrierless amplitude and phase modulation (CAP) signals in FSO channels is prone to producing obvious nonlinearity effects and inter-symbol interference (ISI), which seriously limit the transmission efficiency of the transmitted information. Many digital signal processing techniques have been proposed to solve this problem [2]. The neural network technology in machine learning is an efficient and accurate equalization technology because of its strong approximation ability and the excellent characteristics whereby it can be processed directly from end to end without starting from theory [3]. It can effectively approximate the transmission function of the backlight channel and offset the nonlinear effect. However, choosing which network to use as an equalizer is often a big problem, because both the accuracy and processing rate should be taken into account. Recurrent neural networks (RNNs) (our study used GRU, a type of RNN) show excellent performance in eliminating nonlinear distortion due to their excellent properties for timing processing [4,5,6]. However, because of its recursive structure, it is difficult to realize parallel processing, resulting in a poor processing rate as an equalizer. Using CNNs, which have a structure that is easy to parallelize, the speed can be greatly improved, but the accuracy is not so ideal [7]. How to balance accuracy and speed was the focus of this research.

We introduce a new way to balance the excellent characteristics of GRUs for timing and the parallelization of CNNs for speed improvement: knowledge distillation (KD). KD is a method of transferring a more complex teacher model to a relatively simple student model, which can approximate or exceed the performance of the teacher model, and enjoy the speed increase from the simplified structure [8]. KD can use the structure of CNNs for parallelization, and it can also allow the CNN to absorb the recognition of time sequence information brought by the GRU. KD in the past usually distills the same kind of network, from complex to simple, but there are few studies on this kind of cross structure at present [9]. In this study, the method of distilling across the network structure was adopted so that the student network CNN can have the advantages of both types of networks and improve the speed by using parallelization rather than reducing the computational complexity. In this way, the CNN equalizer after KD from the biGRU can be used to quickly and accurately complete the signal recovery work in the hardware. In particular, in bandwidth-limited systems like the one in our experiments, a network architecture that can be quickly trained for minimalist deployment is cost-effective. In this work, we accomplished the following:

1. We propose a solution using KD to distill the biGRU-based equalizer to obtain a network structure that can be parallelized. We transfer the knowledge that can recognize temporal signals from the teacher model based on the biGRU to the student model 1D-CNN, which can be processed in parallel, so that the student model also has the ability to recognize temporal information [10]. We compare the biGRU, 1D-CNN after KD and 1D-CNN without KD in terms of Q-factor and equalization velocity. The experimental data showed that the Q-factor of the 1D-CNN increased by 1 dB after KD learning from the biGRU, and KD increased the RoP sensitivity of the 1D-CNN by 0.89 dB on the HD-FEC threshold of 1 × 10^−3^.
2. More importantly, we used the parallelization ability of the student model to achieve a huge speed increase: the proposed 1D-CNN reduces the computational time by 97% and the number of trainable parameters by 99.3% compared with the biGRU.
3. Through experimentation, the effectiveness of the network after KD was tested in different situations with different parameters. The results demonstrate that the proposed minimalist 1D-CNN equalizer holds significant promise for future practical deployments in optical wireless communication systems.

The rest of this paper is organized as follows: the second section introduces the background and method of KD and the characteristics of GRUs and CNNs, and explains the advantages of GRUs for timing information and the advantages of CNN parallelization; the third section introduces the setup of the experiment and the training of a real network; the fourth section introduces a series of comparisons and an analysis of the network performance; and the fifth section is the summary of this paper.

## 2. Methods

### 2.1. Knowledge Distillation

Knowledge distillation is a model compression method that is a training method based on the “teacher-student network idea”, which introduces soft targets associated with the teacher network (complex, but with superior predictive accuracy) as part of the overall loss to induce training of the student network (lean, low complexity, better suited for inference deployment) to achieve knowledge transfer [11]. In past research, KD has often been used to complete classification tasks [12,13].

**Figure 1:**
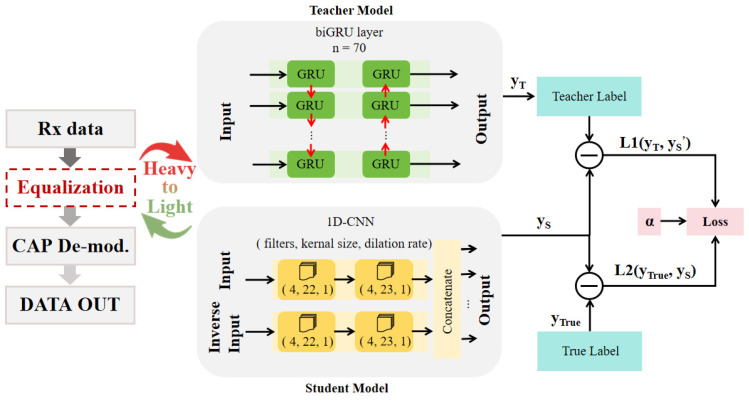
Structure of KD with biGRU as the teacher model and 1D-CNN as the student model to construct a minimalist deployment of a neural network equalizer.

The task completed by KD in this paper is a regression task. Although KD has rarely been used for regression in past studies, there are some results from regression tasks performed by KD which also provide some basis for our research [9]. The structure of our KD is roughly shown in Figure 1. The teacher model is a biGRU of which the output we have softened as *y_T_*, and the student model is a 1D-CNN of which the output is *y_S_* without any processing. After this, we put *y_T_* and the similarly softened *y_S_*′ into a loss function, and *y_S_* and the true label *y_true_* into another loss function. The two are weighted by α and added together to obtain the total loss, as shown in the Formula (1). Training the student model according to this total loss can make the student model learn from both the teacher label and the real label, and obtain the common information of both. α controls the proportion of the student model learning from the two labels. A larger alpha indicates a greater knowledge contribution from the teacher model. In addition, it is worth noting that the loss function in the formula can use multiple parameters, such as the Euclidean distance, mean square error and cross entropy; the Euclidean distance is used in this paper because it reflects the best performance.

(1) $$L_{\mathrm{KD}}=\alpha L({y_{S}}^{\prime },y_{T})+(1-\alpha )L(y_{S},y_{true})$$

Therefore, under the action of KD, our student model, 1D-CNN, can learn part of the knowledge of the teacher’s model, biGRU, especially the advantages of time sequence information mentioned above. In this way, the parallelized 1D-CNN can be skillfully used to greatly reduce the processing time of the equalizer. As such, we can change a heavy teacher model biGRU into a light student model 1D-CNN, which is a practical way to construct the minimalist deployment of a neural network equalizer.

### 2.2. GRU

Recurrent neural networks (RNNs) are a class of neural networks specifically designed to process samples of timing data. If the input information contains time-dependent information, the RNN will produce better results than other neural networks [14].

**Figure 2:**
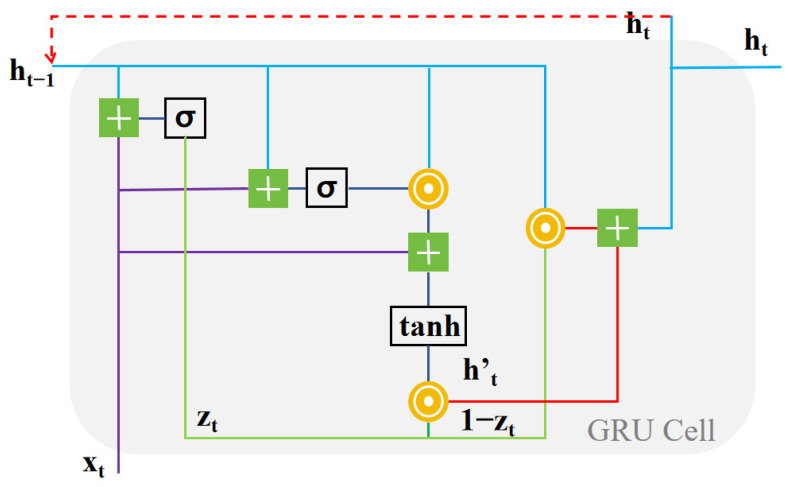
Structure of a GRU cell.

A gated recurrent unit (GRU) is an RNN variant that was originally designed to solve the problem of disappearing gradients in standard RNNs [15]. The structure is shown in Figure 2.

The basic structure of a GRU determines that it has a good performance in processing timing information. The experimental system used in this paper is based on the signal modulated by CAP. In order to demodulate the two IQ channels of the CAP data correctly, the time domain response peak of the matching filter at the receiving end must appear in its pulse center, which means that an accurate sampling clock must be set for CAP demodulation. However, in the actual system, as long as there is a slight sampling clock deviation at the receiving end, it will result in serious inter-code crosstalk to the CAP signal. This kind of inter-code crosstalk contains timing information, so using a GRU to equalize the processing will have a better effect, which was also verified by the experiments (see Section 5).

More specifically, a biGRU was used in this study. biGRUs use a forward and reverse two-layer GRU structure, which helps to analyze timing information from both directions and achieve better equalization [16].

### 2.3. CNN and Parallelization

**Figure 3:**
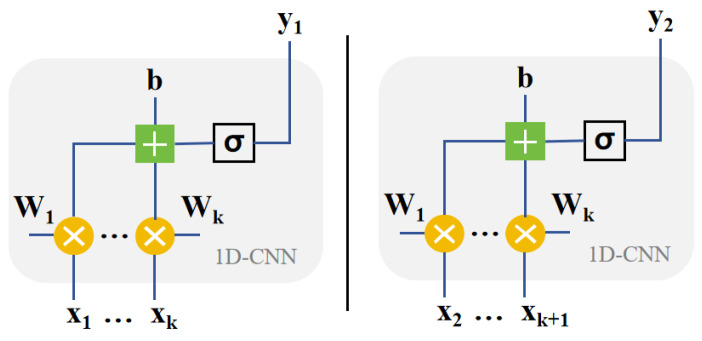
Structure of the 1D-CNN cell.

In this study, we chose 1D-CNN as the student model. The structure of the 1D-CNN is shown in Figure 3. Unlike a GRU, a 1D-CNN is a feedforward neural network in which each unit separately processes information about the current moment [7,17]. In other words, it does not respond well to correlations between moments. The 1D-CNN in Formula (2) shows that *ϕ* is the nonlinear activation function, *x* is the input, *k* is the weight and *b* is the bias. Obviously, it only responds to the current input. However, CNNs have some advantages over other parallelizable feedforward neural networks, such as MLPs, because MLPs lack design time processing and do not take into account the sequence of data points, which limits their ability to effectively model the dynamics of time series.

(2) $$y_{i}^{f}=\phi (\sum_{n=1}^{n_{i}}\sum_{j=1}^{n_{k}}x_{i+j-1,n}^{in}\cdot k_{j,n}^{f}+b^{f})$$

However, since each structure processes information individually, without the need for recursive operations such as GRUs, 1D-CNNs provide the conditions for parallelization. The GRU cannot be parallelized because it requires recursion, and each moment requires information from the previous moment. In computers, a network structure that can be parallelized can make more efficient use of the computing power of the CPU or GPU, and accelerate the training and use of neural networks. Parallelization also plays an extremely important role in practical applications: parallel computing improves computational efficiency and reduces the time required for equalizers to process information. More importantly, parallelization allows for neural network-based equalizers to be applied in real hardware. In particular, in optical networks, high-speed data processing and latency can be a key factor in determining the equalizer’s performance.

## 3. Experiment Setup and Network Training

### 3.1. Experimental System Setup

**Figure 4:**
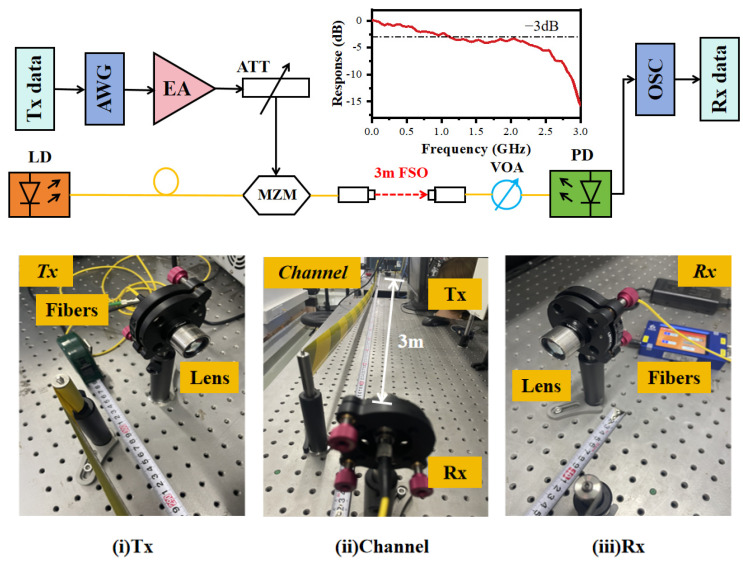
Experimental setup and the actual picture of the experiment.

The detailed experimental setup of our FSO system, as illustrated in Figure 4, is delineated herein. At the transmitter side, the digital signal is subjected to the arbitrary waveform generator (AWG, KEYSIGHT MI9505A). Subsequently, the transmitted signal is sent into an electronic amplifier (EA) and an attenuator (ATT). The modulated signal is then channeled to the laser via a Mach–Zehnder modulator (MZM). A collimating lens is employed to focus the emitted light. The channel encompasses a 3-m expanse of free space. The frequency response diagram of the channel is delineated in Figure 4. At the receiver side, the received light is initially converged by another collimating lens before traversing an optical attenuator (OA) and a photodetector (PD, Agilent 11982A). The signal is then sampled with an oscilloscope (OSC, KEYSIGHT MXR604A). Essential received data, imperative for subsequent neural network training and testing, are seriously archived for comprehensive analysis.

The digital signal processing is as follows. First, a pseudo-random bit sequence (PRBS) is mapped into 32 QAM complex symbols. These symbols are then subjected to up-sampling by a factor of 4, followed by their division into in-phase (I) and quadrature (Q) components. Subsequent application of pulse-shaping filters to both components lead to their amalgamation, followed by power normalization to yield the transmitted data. Upon reception, the received digital signal, Tx, undergoes equalization of our designed equalizer. Following this, CAP demodulation is employed to demodulate the equalized signal. The resultant signal is then down-sampled and subsequently de-mapped after symbol-wise equalization, thus facilitating the recovery of the original data.

### 3.2. Network Training

In the network training, since we needed to compare the teacher network (biGRU), the undistilled student network and the distilled student network (1D-CNN), we first trained the biGRU to obtain an optimal teacher network. After this, the 1D-CNN was trained using KD, and a 1D-CNN with the same network structure was trained simultaneously without KD. The performance of the three networks was compared after the training was completed. We used the signal after the channel as the input, and the initial signal (both 49,152 × 1) as the label for training. The batch size was 256. We used 30% of the signal as the training set, and 70% as the test set.

**Figure 5:**
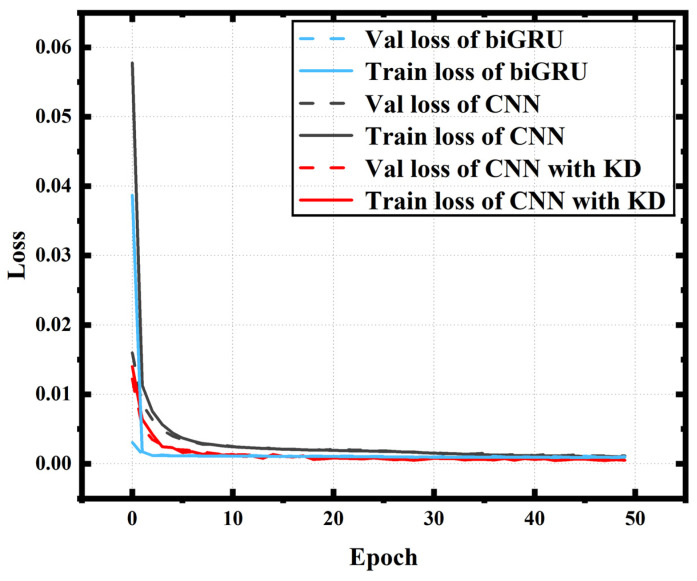
The loss curve of the teacher model biGRU, 1D-CNN and 1D-CNN with KD.

Teacher model: The teacher model is a biGRU, and its structure is shown in Figure 1. The purpose of training the teacher model is firstly to create teacher labels for the subsequent KD and secondly to prepare for the subsequent performance comparisons. The biGRU layer has 70 hidden units (n = 70), the loss function used in this model uses the mean square error (MSE), the optimizer is Adam and the learning rate is 10^−3^. The input window has a size of 47 input symbols, which are used together to recover one symbol. The teacher model was trained with 50 epochs and the loss curve is shown in Figure 5.

Student model: In order to better learn the performance of the teacher model biGRU, the student model 1D-CNN that we adopted requires some features. First of all, since the teacher model is bidirectional and can learn both forward and reverse time series information, we also adopted a two-branch 1D-CNN structure, with one branch processing forward information and the other processing reverse information. We also verified that this structure would have better learning performance in the experiment. Secondly, we adopt a 1D-CNN structure with a dilation rate of 1. This is because a model with a dilated CNN structure can better learn the processing power of biGRU for time series information [18]. The convolution kernel will have a larger perceptual field of view, which helps the 1D-CNN to learn longer time series information [19]. The various parameters of a 1D-CNN are obtained by traversing the parameters through Bayesian optimization. Two layers of a 1D-CNN structure are used in each direction; the structural parameters of the first layer (4, 22, 1) mean that there are 4 filters with a kernel size of 22 and a dilation rate of 1, while the second layer (4, 23, 1) means that there are 4 filters with a kernel size of 23 and a dilation rate of 1. The activation function of 1D-CNN is Relu [20]. The training takes an alpha value of 0.3, which is the alpha value with the best performance by traversing. The same student model was also trained with 50 epochs, and the loss curve is shown in Figure 5.

Student model without KD: This model directly uses the student model structure under KD to better compare the performance improvement brought by KD. In order to be as consistent as possible with the previous network training, the loss function used in the model is the mean square error (MSE), the optimizer is Adam, and the input window has a size of 47 input symbols, which are collectively used to recover one symbol. The model was similarly trained with 50 epochs and the loss curve is shown in Figure 5.

## 4. Results and Discussion

### 4.1. The Role of Knowledge Distillation

**Figure 6:**
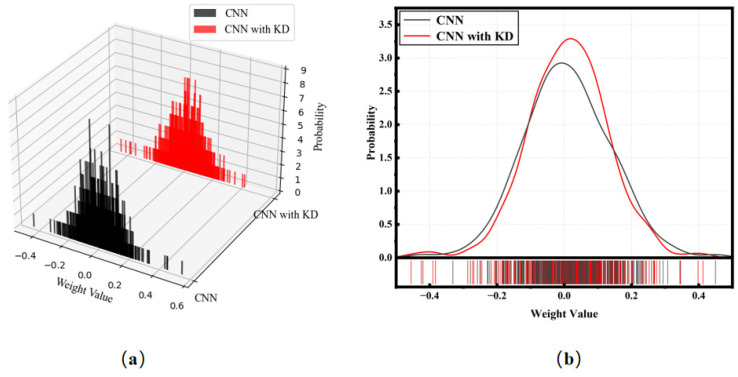
(**a**) Comparison of the weight distributions of 1D-CNN and 1D-CNN with KD. (**b**) Intuitional comparison of the weight distributions of 1D-CNN and 1D-CNN with KD.

The results showed that the 1D-CNN had a better performance after KD. For the same network structure, the impact of KD may be reflected in the weight distribution. Therefore, in Figure 6, we show the weight distribution of the 1D-CNN with and without KD. In Figure 6b, it can be intuitively seen that KD brings a more regularized weight distribution to the 1D-CNN. Such weights will effectively reduce overfitting. It can be inferred that the label of the teacher model improves the performance of the student model by regularizing the weight distribution.

### 4.2. Equalization Performance

We compared the performance of the teacher model (biGRU), student model (1D-CNN) and 1D-CNN without KD but with the same structure with different experimental parameters (VPP, baud rate and RoP) and used the Q-factor to measure the performance.

**Figure 7:**
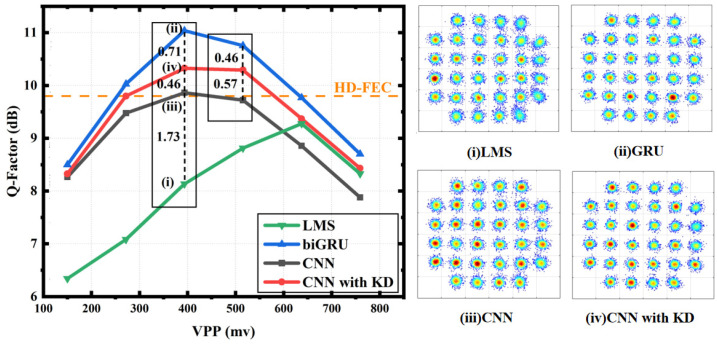
Q-factor comparison and constellation diagram of the LMS, biGRU, 1D-CNN and 1D-CNN with KD with a changing VPP.

As shown in Figure 7, for the VPP, all three achieved the best performance at 400 mv, and the 1D-CNN network with KD had a 0.46 dB improvement in the Q-factor compared with the network without KD, and also had a 0.71 dB gap compared with the teacher network biGRU. In addition, at 500 mv, no KD had the most significant effect on the 1D-CNN. Here, KD produced a 0.57 dB improvement to the 1D-CNN and only a 0.46 dB difference compared to the teacher network. When the VPP is small, the difference between the three was not so obvious, which may be caused by the small influence of the timing information on the equilibrium. However, in the process of the continuous improvement of the VPP, KD maintained the improvement of the effect on the 1D-CNN. For the HD-FEC (BER = 1 × 10^−3^) threshold [21], the 1D-CNN without KD only exceeded the threshold at 400 mv, while the 1D-CNN after KD crossed the threshold in the range of 250–550 mv, showing the significant improvement brought by KD. In addition, we compared the NN algorithm with the LMS algorithm, and we verified the ability of the neural network to function as a nonlinear equalizer.

**Figure 8:**
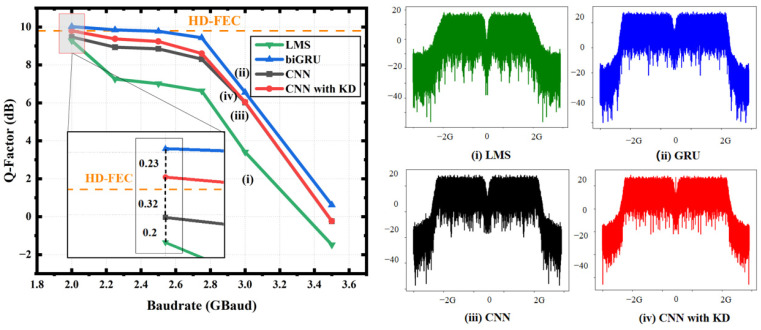
Q-factor comparison and spectrum diagram of the LMS, biGRU, 1D-CNN and 1D-CNN with KD with a changing baud rate.

The baud rate results are shown in Figure 8; it is obvious that the lower the baud rate, the better the performance of each network. However, it is worth noting that when the baud rate was 2 GBaud, the Q-factor for the 1D-CNN with KD increased by 0.32 dB, and the Q-factor difference between the student network and the teacher network after KD was only 0.23 dB. Importantly, only with KD could the 1D-CNN exceed the threshold when the baud rate was 2 Gbaud. When the baud rate was lower than 3 GBaud, KD always brought about performance gains for the student model, but there was no effect of KD on the teacher model. It is speculated that the network of the student model at this time cannot always absorb knowledge from the teacher model in the KD, and it may be necessary to change the network architecture to continue learning. In addition, we show the spectrum diagram of the signal at 3 GBaud for all the algorithms in Figure 8, which shows the equalization performance in a bandwidth-limited system in our experiment.

**Figure 9:**
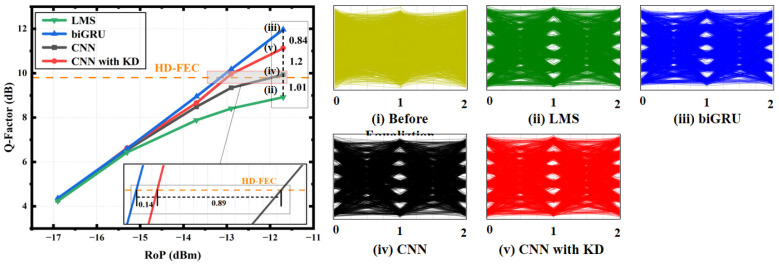
Q-factor comparison of the LMS, biGRU, 1D-CNN and 1D-CNN with KD with a changing RoP, and the eye diagrams of the in-phase signal before and after equalization.

Finally, for the receiving optical power RoP (shown in Figure 9), when the RoP is low (less than −15.8 dB), the performances of the student model, teacher model and student model without KD were almost the same, which may be because the time series information had little effect on the signal recovery at this time. In the process of the gradual increase in RoP later on, KD again manifested its role, and when the RoP reached its maximum, KD brought about a Q-factor improvement for the student model 1D-CNN (up to 1.2 dB), which was the largest improvement among all the experiments. At this time, the gap between the student model and the teacher model after KD was 0.84 dB. Most importantly, KD increased the RoP of the 1D-CNN by 0.89 dB on the HD-FEC threshold, which is enough to show that the role of KD is very significant. The eye diagrams of the in-phase signal before and after equalization demonstrate the existence of inter symbol interference and its suppression after equalization.

These experiments verified that KD does improve the equalization performance of the student model. Although there were still some gaps between the student model and the teacher model, we will analyze the huge improvement in time of the student model in the next section. Under comprehensive consideration, the student model after KD is the optimal solution.

### 4.3. Speed Performance

In order to obtain an equalizer with the best comprehensive properties, the equalization speed should be considered in addition to the equalization performance. As mentioned earlier, only a high-speed equalizer can be used with actual hardware. This is why a 1D-CNN, which can parallelize, was chosen as the student model. Therefore, we also conducted experiments to record the time for the teacher and student networks to complete signal equalization. Since KD does not change the reasoning time of the same network structure, we only needed to compare the teacher and student networks, not the two networks that undergo KD or not. Using the same CPU, the time for the teacher network and the student network to complete signal equalization is shown in Table 1 below. The average equalization time for the student model 1D-CNN was 0.49 s, while the average equalization time for the teacher model biGRU was 14.36 s. Compared with the biGRU, the proposed 1D-CNN equalizer reduced the computational time consumption by 97%, with only a 0.5 dB Q-factor penalty. This also verifies the theory that the parallel structure of a 1D-CNN can greatly reduce inference delay compared with the recursive structure of a biGRU. In addition, the 1D-CNN only used 0.7% of the number of trainable parameters as biGRU, which means a far lower computation complexity and shorter training time.

It can be seen that the equalization time of the 1D-CNN structure was only 3% that of biGRU. Although the biGRU had a better equalization performance than the 1D-CNN, the equalization performance of the 1D-CNN greatly improved after KD, reaching the same level as that of the biGRU. Thus, such a large rate improvement brought about by parallelization makes the 1D-CNN after KD the best choice of equalizer for minimalist deployment.

## 5. Conclusions

This paper presents a technique for transferring the knowledge of a biGRU equalizer to a parallelizable 1D-CNN equalizer using KD. This approach enables the parallelization of signal processing, allowing us to essentially simplify the hardware implementation of neural network models. In addition, the characteristics of the KD method that help students learn and the limitations of KD were emphasized. Using KD, we enhanced the 1D-CNN’s ability to process timing signals to improve its performance: the Q-factor of the 1D-CNN increased by 1 dB after KD learning from the biGRU, and KD increased the RoP sensitivity of the 1D-CNN by 0.89 dB with the HD-FEC threshold. We also show that, compared with the original biGRU model, the proposed KD feedforward equalizer significantly reduced the signal processing delay: the proposed 1D-CNN equalizer reduced the computational time consumption by 97% and the number of trainable parameters by 99.3%, with only a 0.5 dB Q-factor penalty compared with the biGRU. These results demonstrate that the proposed minimalist 1D-CNN equalizer holds significant promise for future practical deployments in optical wireless communication systems.

## Figures and Tables

**Table 1:** The performance comparison in terms of the speed between the biGRU and 1D-CNN with KD.

	Number of Trainable Parameters	Time per Process (s)
1D-CNN with KD	961	0.49
biGRU	126,281	14.36

## Data Availability

The data that support the findings of this study are available from the corresponding author upon reasonable request.
